# Statistical analysis of dependent competing risks model from Gompertz distribution under progressively hybrid censoring

**DOI:** 10.1186/s40064-016-3421-9

**Published:** 2016-10-07

**Authors:** Yimin Shi, Min Wu

**Affiliations:** Department of Applied Mathematics, Northwestern Polytechnical University, Xi’an, China

**Keywords:** Dependent competing risks model, Gompertz distribution, Progressively hybrid censoring, Bootstrap method

## Abstract

Previous studies have mostly considered the competing risks to be independent even when the interpretation of the failure modes implies dependency. This paper studies the dependent competing risks model from Gompertz distribution under Type-I progressively hybrid censoring scheme. We derive the maximum likelihood estimations of the model parameters, and then the asymptotic likelihood theory and Bootstrap method are used to obtain the confidence intervals. The simulation results are provided to investigate the effects of different dependence structures on the estimations of parameters. Finally, one data set was used for illustrative purpose.

## Background

The competing risks model involves multiple failure modes when only the smallest failure time and the associated failure mode are observed. This model is widely studied in the medical, actuarial, biostatistics and so on, under the assumption of independent competing risks. It is common that a failure is associated with one of the several competing failure modes. Previous studies have mostly considered the competing failure modes to be independent even when the interpretation of the failure modes implies dependency. Such as, in the study of colon cancer, the failure causes were cancer recurrence or death, obviously, such failure causes were dependent [see Lin et al. (1999)]. The competing risks model assuming independence among competing failure modes has been widely studied [see, e.g., Crowder (2001)]. Kundu et al. (2004) analyzed the progressively censored competing risks data, Sarhan (2007) analyzed the competing risks models with generalized exponential distributions, Cramer and Schmiedt (2011) studied the progressively censored competing risks data with Lomax distribution, other related works see, Bunea and Mazzuchi (2006); Balakrishnan and Han (2008); Pareek et al. (2009); Xu and Tang (2011), and so on.

The competing risks model under the assumption of dependent competing failure modes has been considered in the early work by Elandt-Johnson (1976). Afterwards, a number of corresponding works have been devoted to the dependent competing risks model. Zheng and Klein (1995) considered the dependence structure between failure modes is represented by an assumed Archimedean copula. Other works see Escarela and Carriere (2003); Kaishev et al. (2007).

In this paper, we present a dependent competing risks model from Gompertz distribution under Type-I progressively hybrid censoring scheme (PHCS). The Gompertz distribution is one of classical mathematical models and was first introduced by Gompertz (1825), which is a commonly used growth model in actuarial and reliability and life testing, and plays an important role in modeling human mortality and fitting actuarial tables and tumor growth. This distribution has been widely used, see, Ali (2010); Ghitany et al. (2014).

The Type-I PHCS was first proposed by Kundu and Joarder (2006) [see also Childs et al. (2008)]. This censoring scheme has been widely used in reliability analysis, see, Chien et al. (2011); Cramer and Balakrishnan (2013). It can be defined as follows: suppose *n* identical units are put to life test with progressive censoring scheme $(r_{1} ,r_{2} , \ldots ,r_{m} ),\;1 \le m \le n$, the experiment is terminated at time $\tau$, where $\tau \in (0,\infty ),\;r_{i} (i = 1, \cdots ,m)$ and *m* are fixed in advance. At the time of the first failure $t_{1} ,\;r_{1}$ of the remaining units are randomly removed, at the time of the second failure $t_{2} ,\;r_{2}$ of the remaining units are randomly removed and so on. If the *m*th failure time $t_{m}$ occurs before time $\tau$, all the remaining units $R_{m}^{*} = n - m - (r_{1} + \cdots + r_{m - 1} )$ are removed and the terminal time of the experiment is $t_{m}$. On the other hand, if the *m*th failure time $t_{m}$ does not occur before time $\tau$ and only *J* failures occur before time $\tau$, where $0 \le J \le m$. Then all the remaining units $R_{J}^{*} = n - J - (r_{1} + \cdots + r_{J} )$ are removed and the terminal time of the experiment is $\tau$. We denote the two cases as

**Case I** $$t_{1} < t_{2} < \cdots < t_{m} ,\;\;\;{\text{if}}\;t_{m} < \tau$$

**Case II** $$t_{1} < t_{2} < \cdots < t_{J} < \tau < t_{J + 1} < \cdots < t_{m} ,\;\;\;{\text{if}}\;t_{m} > \tau$$

The rest of the paper is organized as follows. “Model description” section provides the model description, “Maximum likelihood estimations (MLEs)” section presents the maximum likelihood estimations of the model parameters. The confidence intervals are provided in “Confidence intervals” section. “Simulation and data analysis” section presents the simulation and data analysis. Conclusion appears in “Conclusion” section.

## Model description

It is assumed that the Gompertz distribution with shape parameter *λ* and scale parameter *θ* has the following probability density function (PDF), cumulative distribution function (CDF) and survival function

1 $$f(t|\lambda ,\theta ) = \theta e^{\lambda t} \exp \{ - (\theta /\lambda )(e^{\lambda t} - 1)\} ,$$

2 $$F(t|\lambda ,\theta ) = 1 - \exp \{ - (\theta /\lambda )(e^{\lambda t} - 1)\} ,$$

3 $$S(t|\lambda ,\theta ) = \exp \{ - (\theta /\lambda )(e^{\lambda t} - 1)\} ,$$

respectively, where $t > 0,\;\lambda > 0,\;\theta > 0$. We denote the Gompertz distribution by $GP(\lambda ,\theta )$.

Suppose variables $Y_{0} ,\;Y_{1} ,\;Y_{2}$ are independent and $Y_{0}$ follows $(\sim )\;GP(\lambda ,\theta_{0} )$, $Y_{1} \sim GP(\lambda ,\theta_{1} )$, $Y_{2} \sim GP(\lambda ,\theta_{2} )$. Define $T_{1} = \hbox{min} (Y_{0} ,Y_{1} )$, $T_{2} = \hbox{min} (Y_{0} ,Y_{2} )$, then the distributions of $T_{1} ,\;T_{2}$ are $GP(\lambda ,\theta_{0} + \theta_{1} )$ and $GP(\lambda ,\theta_{0} + \theta_{2} )$, respectively.

### **Theorem 1**

*The joint survival function of* $(T_{1} ,\;T_{2} )$ *is*

$$\begin{aligned} S_{{T_{1} ,\;T_{2} }} (t_{1} ,t_{2} ) &=& \left\{ {\begin{array}{*{20}l} {S(t_{1} |\lambda ,\theta _{0} + \theta _{1} )S(t_{2} |\lambda ,\theta _{2} )} &\quad {{t}_{{1}} {> t}_{{2}} } \\ {S(t_{1} |\lambda ,\theta _{1} )S(t_{2} |\lambda ,\theta _{0} + \theta _{2} )} &\quad {{t}_{{1}} {{ < t}}_{{2}} } \\ {S(t|\lambda ,\theta _{0} + \theta _{1} + \theta _{2} )} &\quad {{t}_{{1}} {= t}_{{2}} {= t}} \\ \end{array} } \right. \end{aligned}$$

*Proof*

$$\begin{aligned} S_{{T_{1} ,\;T_{2} }} (t_{1} ,t_{2} ) &= P(T_{1} > t_{1} ,T_{2} > t_{2} ) \\ &= P(Y_{0} > \text{max} (t_{1} ,t_{2} ),Y_{1} > t_{1} ,Y_{2} > t_{2} ) \\ &= S(\text{max} (t_{1} ,\;t_{2} )|\lambda ,\theta_{0} )S(t_{1} |\lambda ,\theta_{1} )S(t_{2} |\lambda ,\theta_{2} ) \\ &= \left\{ {\begin{array}{*{20}l} {S(t_{1} |\lambda ,\theta _{0} + \theta _{1} )S(t_{2} |\lambda ,\theta _{2} )} &\quad {{t}_{{1}} { > t}_{{2}} } \\ {S(t_{1} |\lambda ,\theta _{1} )S(t_{2} |\lambda ,\theta _{0} + \theta _{2} )} &\quad {{t}_{{1}} { < t}_{{2}} } \\ {S(t|\lambda ,\theta _{0} + \theta _{1} + \theta _{2} )} &\quad {{t}_{{1}} { = t}_{{2}} { = t}} \\ \end{array} } \right. \end{aligned}$$

### **Corollary 1**

*The joint PDF of* $(T_{1} ,\;T_{2} )$ *can be written as*

$$f_{{T_{1} ,\;T_{2} }} (t_{1} ,t_{2} ) = \left\{ \begin{array} {l} f_{1} (t_{1} ,t_{2} ) \hfill \\ f_{2} (t_{1} ,t_{2} ) \hfill \\ f_{0} (t) \hfill \\ \end{array} \right. = \left\{ \begin{array}{ll} f(t_{1} |\lambda ,\theta_{0} + \theta_{1} )f(t_{2} |\lambda ,\theta_{2} )&\quad t_{1} > t_{2} \\ f(t_{1} |\lambda ,\theta_{1} )f(t_{2} |\lambda ,\theta_{0} + \theta_{2} )&\quad t_{1} < t_{2} \\ \;(\theta_{0} /(\theta_{0} + \theta_{1} + \theta_{2} ))f(t|\lambda ,\theta_{0} + \theta_{1} + \theta_{2} )&\quad t_{1} = t_{2} = t \\ \end{array} \right.$$

*Proof* For the cases $t_{1} > t_{2}$ and $t_{1} < t_{2}$, $f_{1} (t_{1} ,t_{2} ),\;\;f_{2} (t_{1} ,t_{2} )$ can be easily obtained by $- \frac{{\partial^{2} S_{{T_{1} ,\;T_{2} }} (t_{1} ,t_{2} )}}{{\partial t_{1} \partial t_{2} }}$. For the case $t_{1} = t_{2} = t$, by the full probability formula, we have the fact that

4 $$\int_{0}^{\infty } {\int_{0}^{{t_{1} }} {f_{1} (t_{1} ,t_{2} )} dt_{2} dt_{1} } + \int_{0}^{\infty } {\int_{0}^{{t_{2} }} {f_{2} (t_{1} ,t_{2} )} dt_{1} dt_{2} } + \int_{0}^{\infty } {f_{0} (t)} dt = 1 ,$$

where

$$\begin{aligned} \int_0^\infty {\int_0^{{t_2}} {{f_2}({t_1},{t_2})} d{t_1}d{t_2}} &= \int_0^\infty {\int_0^{{t_1}} {({\theta _0} + {\theta _1}){\theta _2}\exp \left\{ {\lambda {t_1} - \frac{{{\theta _0} + {\theta _1}}}{\lambda }({e^{\lambda {t_1}}} - 1)} \right\}} } \\ & \quad \times \exp \left\{ {\lambda {t_2} - \frac{{{\theta _2}}}{\lambda }({e^{\lambda {t_2}}} - 1)} \right\}d{t_2}d{t_1} \\ & = ({\theta _0} + {\theta _1})\int_0^\infty \left[ {\exp \left\{ {\lambda t - \frac{{{\theta _0} + {\theta _1}}}{\lambda }({e^{\lambda t}} - 1)} \right\} - \exp \left\{ {\lambda t - \frac{{{\theta _0} + {\theta _1} + {\theta _2}}}{\lambda }({e^{\lambda t}} - 1)} \right\}} \right] \end{aligned}$$

$$\begin{aligned} \int_0^\infty {\int_0^{{t_2}} {{f_2}({t_1},{t_2})} d{t_1}d{t_2}} &= \int_0^\infty {\int_0^{{t_2}} {{\theta _1}({\theta _0} + {\theta _2})\exp \left\{ {\lambda {t_1} - \frac{{{\theta _1}}}{\lambda }({e^{\lambda {t_1}}} - 1)} \right\}} } \\ &\quad \times \text{exp} \left\{ {\lambda {t_2} - \frac{{{\theta _0} + {\theta _2}}}{\lambda }({e^{\lambda {t_2}}} - 1)} \right\}d{t_1}d{t_2} \\ & = ({\theta _0} + {\theta _2})\int_0^\infty \left[ \exp \left\{ {\lambda t - \frac{{{\theta _0} + {\theta _2}}}{\lambda }({e^{\lambda t}} - 1)} \right\} \right. \\ & \quad\left. - \exp \left\{ {\lambda t - \frac{{{\theta _0} + {\theta _1} + {\theta _2}}}{\lambda }({e^{\lambda t}} - 1)} \right\} \right] dt, \end{aligned}$$

So from (4), we have

$$\begin{aligned} \int_{0}^{\infty } {f_{0} (t)} dt &= 1 - \int_{0}^{\infty } {\int_{0}^{{t_{1} }} {f_{1} (t_{1} ,t_{2} )} dt_{2} dt_{1} } - \int_{0}^{\infty } {\int_{0}^{{t_{2} }} {f_{2} (t_{1} ,t_{2} )} dt_{1} dt_{2} } \\ &= 1 + (2\theta_{0} + \theta_{1} + \theta_{2} )\int_{0}^{\infty } {\exp \left\{ {\lambda t - \frac{{\theta_{0} + \theta_{1} + \theta_{2} }}{\lambda }(e^{\lambda t} - 1)} \right\}dt} \\ & \quad - (\theta_{0} + \theta_{1} )\int_{0}^{\infty } {\exp \left\{ {\lambda t - \frac{{\theta_{0} + \theta_{1} }}{\lambda }(e^{\lambda t} - 1)} \right\}} dt - (\theta_{0} + \theta_{2} )\int_{0}^{\infty } {\exp \left\{ {\lambda t - \frac{{\theta_{0} + \theta_{2} }}{\lambda }(e^{\lambda t} - 1)} \right\}} dt \\ &= \frac{{\theta_{0} }}{{\theta_{0} + \theta_{1} + \theta_{2} }} \hfill \\ \end{aligned}$$

So we have $f_{0} (t) = \frac{{\theta_{0} }}{{\theta_{0} + \theta_{1} + \theta_{2} }}f(t|\lambda ,\theta_{0} + \theta_{1} + \theta_{2} )$. □

**Fig. 1:**
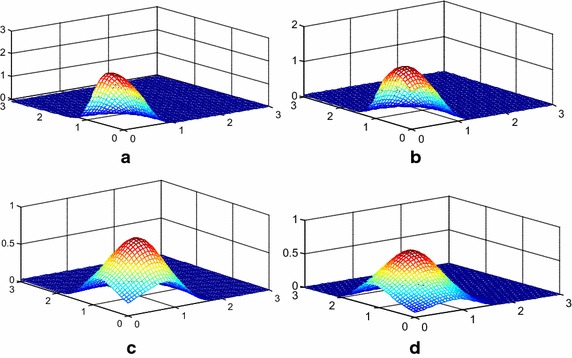
The surface plot of the joint PDF of $T_{1} ,\;T_{2}$ with different values of $\theta_{0} ,\;\theta_{1} ,\;\theta_{2}$ when $\lambda = 2$. **a** $\theta_{0} = \theta_{1} = \theta_{2} = 1$, **b** $\theta_{0} = 0.5,\theta_{1} = \theta_{2} = 0.8$, **c** $\theta_{0} = 0,\theta_{1} = \theta_{2} = 0.4$, **d** $\theta_{0} = \theta_{1} = \theta_{2} = 0.2$

Figure 1 presents the surface plot of $f_{{T_{1} ,\;T_{2} }} (t_{1} ,t_{2} )$ for different values of $\lambda ,\;\theta_{0} ,\;\theta_{1} ,\;\theta_{2}$, from Fig. 1, we can see that the $f_{{T_{1} ,\;T_{2} }} (t_{1} ,t_{2} )$ is unimodal. Define $X = \hbox{min} (T_{1} ,T_{2} )$, and the distribution of $X$ is $GP(\lambda ,\theta_{0} + \theta_{1} + \theta_{2} )$. $\theta_{0} = 0$ indicates that $T_{1} ,\;T_{2}$ are independent. Therefore, $\theta_{0}$ can be regarded as the dependence structure between $T_{1} ,\;T_{2}$.

### Competing risks model

Consider two competing failure modes with latent lifetimes $T_{1} ,T_{2}$ in the experiment under Type-I PHCS, the failure of an individual is caused by any single one of the two failure modes, obviously, the actual lifetime span is $X = \hbox{min} (T_{1} ,T_{2} )$. Let *r* denotes the number of failures that occur before time *τ*, τ* denotes the terminal time. Then, at time all the remaining $R_{r}^{*} = n - r - \sum\nolimits_{l = 1}^{r} {r_{l} }$ units are removed and the experiment is terminated, where $r = m$, $\tau^{*} = t_{r}$, r_m_ = 0 in Case I and *r* = *J*, $\tau^{*} = \tau$ in Case II.

For the competing risks model under Type-I PHCS, $(x_{1} ,\alpha_{1} ),\;(x_{2} ,\alpha_{2} ),\; \ldots ,\;(x_{r} ,\alpha_{r} )$ are the observed failure data, where $x_{1} ,x_{2} , \ldots ,x_{r}$ are order statistics, $\alpha_{l}$ takes any integer in the set $\{ 0,1,2\}$. For $j = 0,1,2$, $$\delta_{j} (\alpha_{l} ) = \left\{ \begin{aligned} 1,\;if\;\alpha_{l} = j \hfill \\ 0,\;if\;\alpha_{l} \ne j \hfill \\ \end{aligned} \right.$$. $n_{0} = \sum\nolimits_{l = 1}^{r} {\delta_{0} (\alpha_{l} )}$ denotes the number of failures caused by the two competing failure modes, $n_{j} = \sum\nolimits_{l = 1}^{r} {\delta_{j} (\alpha_{l} ),} \;j = 1,2$ denotes the number of failures caused by competing failure mode $j\;(j = 1,2)$, where $r = \sum\nolimits_{j = 0}^{2} {n_{j} }$.

## Maximum likelihood estimations (MLEs)

The likelihood function for the two competing risks model under Type-I PHCS can be written as

5 $$\begin{aligned} L(\lambda ,\theta_{0} ,\theta_{1} ,\theta_{2} ) \propto \prod\limits_{l = 1}^{r} {\left\{ {\left( {f_{{T_{1} ,\,\,T_{2} }} (x_{l} ,x_{l} )} \right)^{{\delta_{0} (\alpha_{l} )}} \prod\limits_{j = 1}^{2} {\left[ { - \frac{{\partial S_{{T_{1} ,\,\,T_{2} }} (t_{1} ,t_{2} )}}{{\partial t_{j} }}|_{{(x_{l} ,x_{l} )}} } \right]^{{\delta_{j} (\alpha_{l} )}} \left( {S_{{T_{1} ,\,\,T_{2} }} (x_{l} ,x_{l} )} \right)^{{r_{l} }} } } \right\}} \hfill \\ \times \left( {S_{{T_{1} ,\,\,T_{2} }} (\tau^{*} ,\tau^{*} )} \right)^{{n - r - \sum\nolimits_{l = 1}^{r} {r_{l} } }} , \hfill \\ \end{aligned}$$

where

$$\begin{aligned} f_{{T_{1} ,\,\,T_{2} }} (x_{l} ,x_{l} ) &= (\theta_{0} /(\theta_{0} + \theta_{1} + \theta_{2} ))f(t|\lambda ,\theta_{0} + \theta_{1} + \theta_{2} ) \\ &= \theta_{0} \exp \left\{ {\lambda x_{l} - ((\theta_{0} + \theta_{1} + \theta_{2} )/\lambda )(e^{{\lambda x_{l} }} - 1)} \right\}, \end{aligned}$$

$$\begin{aligned} - \frac{{\partial S_{{T_{1} ,\,\,T_{2} }} (t_{1} ,t_{2} )}}{{\partial t_{1} }}|_{{(x_{l} ,x_{l} )}} &= f(x_{l} |\lambda ,\theta_{1} )S(x_{l} |\lambda ,\theta_{0} + \theta_{2} ) \\ &= \theta_{1} \exp \left\{ {\lambda x_{l} - ((\theta_{0} + \theta_{1} + \theta_{2} )/\lambda )(e^{{\lambda x_{l} }} - 1)} \right\}, \end{aligned}$$

$$\begin{aligned} - \frac{{\partial S_{{T_{1} ,\,\,T_{2} }} (t_{1} ,t_{2} )}}{{\partial t_{2} }}|_{{(x_{l} ,x_{l} )}} &= f(x_{l} |\lambda ,\theta_{2} )S(x_{l} |\lambda ,\theta_{0} + \theta_{1} ) \\ &= \theta_{2} \exp \left\{ {\lambda x_{l} - ((\theta_{0} + \theta_{1} + \theta_{2} )/\lambda )(e^{{\lambda x_{l} }} - 1)} \right\}, \end{aligned}$$

$$\eqalign{ & {S_{{T_1},{\kern 1pt} {\kern 1pt} {T_2}}}({x_l},{x_l}) = S({x_l}|\lambda ,{\theta _0} + {\theta _1} + {\theta _2}) \cr & \qquad \qquad \quad = {\text{exp}}\{ - (({\theta _0} + {\theta _1} + {\theta _2})/\lambda )({e^{\lambda {x_l}}} - 1)\} \cr}$$

$$\begin{aligned} S_{{T_{1} ,\,\,T_{2} }} (\tau^{*} ,\tau^{*} ) &= S(\tau^{*} |\lambda ,\theta_{0} + \theta_{1} + \theta_{2} ) \\ &= \exp \left\{ { - ((\theta_{0} + \theta_{1} + \theta_{2} )/\lambda )(e^{{\lambda \tau^{*} }} - 1)} \right\}. \end{aligned}$$

So the likelihood function can be written as

6 $$\begin{aligned}L(\lambda ,\theta_{0} ,\theta_{1} ,\theta_{2} ) & \propto \left( {\prod\limits_{j = 0}^{2} {\theta_{j}^{{n_{j} }} } } \right)\exp \left\{ \lambda \sum\limits_{l = 1}^{r} x_{l} - ((\theta_{0} + \theta_{1} + \theta_{2} )/\lambda ) \right.\\&\quad\times \left.\left[ \sum\limits_{l = 1}^{r} {(r_{l} + 1)(e^{{\lambda x_{l} }} - 1) + (n - r - \sum\limits_{l = 1}^{r} {r_{l} } )(e^{{\lambda \tau^{*} }} - 1)} \right] \right\} \end{aligned}.$$

By setting the first partial derivative of $\log L$ about $\theta_{0} ,\theta_{1} ,\theta_{2} ,\lambda$ to zero, we get

7 $$\frac{\partial \log L}{{\partial \theta_{0} }} = n_{0} /\theta_{0} - (1/\lambda )\left[ {\sum\limits_{l = 1}^{r} {(r_{l} + 1)(e^{{\lambda x_{l} }} - 1)} + (n - r - \sum\limits_{l = 1}^{r} {r_{l} } )(e^{{\lambda \tau^{*} }} - 1)} \right] = 0,$$

8 $$\frac{\partial \log L}{{\partial \theta_{1} }} = n_{1} /\theta_{1} - (1/\lambda )\left[ {\sum\limits_{l = 1}^{r} {(r_{l} + 1)(e^{{\lambda x_{l} }} - 1)} + (n - r - \sum\limits_{l = 1}^{r} {r_{l} } )(e^{{\lambda \tau^{*} }} - 1)} \right] = 0,$$

9 $$\frac{\partial \log L}{{\partial \theta_{2} }} = n_{2} /\theta_{2} - (1/\lambda )\left[ {\sum\limits_{l = 1}^{r} {(r_{l} + 1)(e^{{\lambda x_{l} }} - 1)} + (n - r - \sum\limits_{l = 1}^{r} {r_{l} } )(e^{{\lambda \tau^{*} }} - 1)} \right] = 0.$$

10 $$\begin{aligned} \frac{\partial \log L}{\partial \lambda } &= \sum\limits_{l = 1}^{r} {x_{l} } + ((\theta_{0} \,+\, \theta_{1} \,+\, \theta_{2} )/\lambda^{2} )\left[ {\sum\limits_{l = 1}^{r} {(r_{l} + 1)(e^{{\lambda x_{l} }} - 1)} + (n - r - \sum\limits_{l = 1}^{r} {r_{l} } )(e^{{\lambda \tau^{*} }} - 1)} \right] \\&\quad- ((\theta_{0} + \theta_{1} + \theta_{2} )/\lambda )\left[ {\sum\limits_{l = 1}^{r} {(r_{l} + 1)x_{l} e^{{\lambda x_{l} }} } + (n - r - \sum\limits_{l = 1}^{r} {r_{l} } )\tau^{*} e^{{\lambda \tau^{*} }} } \right] = 0 .\end{aligned}$$

From (7), (8) and (9), the estimates of $\theta_{j} ,j = 0,1,2$ are given by

11 $$\hat{\theta }_{j} (\lambda ) = n_{j} \lambda /\left[ {\sum\limits_{l = 1}^{r} {(r_{l} + 1)(e^{{\lambda x_{l} }} - 1)} + (n - r - \sum\limits_{l = 1}^{r} {r_{l} } )(e^{{\lambda \tau^{*} }} - 1)} \right] .$$

Substituting $\hat{\theta }_{j} (\lambda )$ into $\log L$ and ignoring the constant, we obtain the profile log-likelihood function of *λ* as

12 $$g(\lambda ) \propto \sum\limits_{j = 0}^{2} {n_{j} \left[ {\ln \lambda - \ln \left( {\sum\limits_{l = 1}^{r} {(r_{l} + 1)e^{{\lambda x_{l} }} } + \left(n - r - \sum\limits_{l = 1}^{r} {r_{l} } \right)e^{{\lambda \tau^{*} }} } \right)} \right]} + \lambda \sum\limits_{l = 1}^{r} {x_{l} } .$$

### **Lemma 1**

*The profile log-likelihood function* $g(\lambda )$ *is concave*.

*Proof* Denote $q(\lambda ) = \sum\nolimits_{l = 1}^{r} {(r_{l} + 1)e^{{\lambda x_{l} }} } + c_{1} e^{{\lambda \tau^{*} }}$, where $c_{1} = n - r - \sum\nolimits_{l = 1}^{r} {r_{l} }$. Therefore, we get $q^{\prime}(\lambda ) = \sum\nolimits_{l = 1}^{r} {(r_{l} + 1)x_{l} e^{{\lambda x_{l} }} } + c_{1} \tau^{*} e^{{\lambda \tau^{*} }} ,$ $q^{''}(\lambda ) = \sum\nolimits_{l = 1}^{r} {(r_{l} + 1)x_{l}^{2} e^{{\lambda x_{l} }} } e^{\lambda x_{l}} +c_{1} \tau ^{{*}2} e^{\lambda \tau^{*}}$

$$q^{\prime\prime}(\lambda )q(\lambda ) - \left( {q'(\lambda )} \right)^{2} \; = \;\sum\limits_{l = 1}^{r} {a_{l}^{2} } \sum\limits_{l = 1}^{r} {b_{l}^{2} } - \left( {\sum\limits_{l = 1}^{r} {a_{l} b_{l} } } \right)^{2} + c_{1} e^{{\lambda \tau^{*} }} \sum\limits_{l = 1}^{r} {(a_{l} - b_{l} \tau^{*} )^{2} } ,$$

where $$a_{l} = (r_{l} + 1)^{1/2} x_{l} e^{{\lambda x_{l} /2}}$$, $$b_{l} = (r_{l} + 1)^{1/2} e^{{\lambda x_{l} /2}}$$.

$q^{\prime\prime}(\lambda )q(\lambda ) - \left( {q'(\lambda )} \right)^{2} \ge 0$ by the Cauchy–Schwarz inequality, therefore $q^{\prime\prime}(\lambda )q(\lambda ) \ge \left( {q'(\lambda )} \right)^{2}$, which implies that the second derivative of $g(\lambda )$ is negative, so $g(\lambda )$ is concave. □

From Lemma 1, we know that $g(\lambda )$ is unimodal and it has a unique maximum. Since $g(\lambda )$ is unimodal, most of the standard iterative procedure can be used to find the MLE. So we propose to use the following simple algorithm. Substituting $\hat{\theta }_{j} (\lambda )$ into (10), the MLE $\hat{\lambda }$ of $\lambda$ satisfies the following equation,

13 $$\lambda = h(\lambda ),$$

where $$h(\lambda ) = 1/\left[ {\frac{{\sum\nolimits_{l = 1}^{r} {(r_{l} + 1)x_{l} e^{{\lambda x_{l} }} } \,+\, c_{1} \tau^{*} e^{{\lambda \tau^{*} }} }}{{\sum\nolimits_{l = 1}^{r} {(r_{l} + 1)(e^{{\lambda x_{l} }} - 1)} \,+\, c_{1} (e^{{\lambda \tau^{*} }} - 1)}} \,-\, \frac{{\sum\nolimits_{l = 1}^{r} {x_{l} } }}{{\sum\nolimits_{j = 0}^{2} {n_{j} } }}} \right].$$

Using the method of a simple iterative scheme proposed in the literature by Kundu (2007), we can solve the shape parameter $\lambda$ from (13). Start with an initial guess of $\lambda$, say $\lambda^{(0)}$, then obtain $\lambda^{(1)} = h(\lambda^{(0)} )$ and proceed in this way to obtain $\lambda^{(n + 1)} = h(\lambda^{(n)} )$. Stop the iterative procedure when $\left| {\lambda^{(n + 1)} - \lambda^{(n)} } \right| < \varepsilon$, some pre-assigned tolerance limit. Once we obtain $\hat{\lambda }$, the MLEs of $\theta_{j} ,j = 0,1,2$ can be obtained from (11) as $\hat{\theta }_{j} ,j = 0,1,2$.

## Confidence intervals

### Observed fisher information

In this section, we will construct the asymptotic confidence intervals (ACIs) for the parameters $\theta_{0} ,\theta_{1} ,\theta_{2} ,\lambda$ using the asymptotic likelihood theory. The observed Fisher information matrix is denoted by

$$I(\theta_{0} ,\theta_{1} ,\theta_{2} ,\lambda ) = \left[ {\begin{array}{*{20}c} {I_{11} } &\quad {I_{12} } &\quad {I_{13} } &\quad {I_{14} } \\ {I_{21} } &\quad {I_{22} } &\quad {I_{23} } &\quad {I_{24} } \\ {I_{31} } &\quad {I_{32} } &\quad {I_{33} } &\quad {I_{34} } \\ {I_{41} } &\quad {I_{42} } &\quad {I_{43} } &\quad {I_{44} } \\ \end{array} } \right]$$,

where the elements of which are negative second partial derivatives of $\log L$.

$$I_{(j + 1)(j + 1)} = - \frac{{\partial^{2} \log L}}{{\partial \theta_{j}^{2} }} = n_{j} /\theta_{j}^{2} ,\quad j = 0,1,2,$$

$$\begin{aligned} I_{44}& = - \frac{{\partial^{2} \log L}}{{\partial \lambda^{2} }} = \left(2\left(\sum\limits_{j = 0}^{2} {\theta_{j} } \right)/\lambda^{3} \right)\left[ {\sum\limits_{l = 1}^{r} {(r_{l} + 1)(e^{{\lambda x_{l} }} - 1)} + c_{1} (e^{{\lambda \tau^{*} }} - 1)} \right] \\ & \quad- \left(2\left(\sum\limits_{j = 0}^{2} {\theta_{j} } \right)/\lambda^{2} \right)\left[ {\sum\limits_{l = 1}^{r} {(r_{l} + 1)x_{l} e^{{\lambda x_{l} }} } + c_{1} \tau^{*} e^{{\lambda \tau^{*} }} } \right] + \left(\left(\sum\limits_{j = 0}^{2} {\theta_{j} } \right)/\lambda \right) [ {(r_{l} + 1)x_{l}^{2}} {(r_{l} + 1)x_{l}^{2}} e^{{\lambda x_{l} }} + c_{1} {\tau}^{*} e ^{{\lambda} {\tau}^{*}} \end{aligned}$$

$$\begin{aligned} I_{(j + 1)4} = I_{4(j + 1)} &= - \frac{{\partial^{2} \log L}}{{\partial \theta_{j} \partial \lambda }} \\ &= - (1/\lambda^{2} )\left[ {\sum\limits_{l = 1}^{r} {(r_{l} + 1)(e^{{\lambda x_{l} }} - 1)} + c_{1} (e^{{\lambda \tau^{*} }} - 1)} \right] \\ &\quad + (1/\lambda )\left[ {\sum\limits_{l = 1}^{r} {(r_{l} + 1)x_{l} e^{{\lambda x_{l} }} } + c_{1} \tau^{*} e^{{\lambda \tau^{*} }} } \right],\quad j = 0,1,2, \end{aligned}$$

$$I_{ij} = I_{ji} = 0,\;\;i = 1,2,3;\;j = i + 1, \ldots ,3.$$

Denote $V$ as the approximate asymptotic variance–covariance matrix of the MLEs of $\theta_{0} ,\theta_{1} ,\theta_{2} ,\lambda$ and $\hat{V}$ as the estimation of $V$, we get

$$\hat{V} = \left[ {\begin{array}{*{20}c} {\hat{V}_{11} } &\quad {\hat{V}_{12} } &\quad {\hat{V}_{13} } &\quad {\hat{V}_{14} } \\ {\hat{V}_{21} } &\quad {\hat{V}_{22} } &\quad {\hat{V}_{23} } &\quad {\hat{V}_{24} } \\ {\hat{V}_{31} } &\quad {\hat{V}_{32} } &\quad {\hat{V}_{33} } &\quad {\hat{V}_{34} } \\ {\hat{V}_{41} } &\quad {\hat{V}_{42} } &\quad {\hat{V}_{43} } &\quad {\hat{V}_{44} } \\ \end{array} } \right] = \left[ {\begin{array}{*{20}c} {\hat{I}_{11} } &\quad {\hat{I}_{12} } &\quad {\hat{I}_{13} } &\quad {\hat{I}_{14} } \\ {\hat{I}_{21} } &\quad {\hat{I}_{22} } &\quad {\hat{I}_{23} } &\quad {\hat{I}_{24} } \\ {\hat{I}_{31} } &\quad {\hat{I}_{32} } &\quad {\hat{I}_{33} } &\quad {\hat{I}_{34} } \\ {\hat{I}_{41} } &\quad {\hat{I}_{42} } &\quad {\hat{I}_{43} } &\quad {\hat{I}_{44} } \\ \end{array} } \right]^{ - 1} .$$

By the asymptotic distribution of MLEs, $(\hat{\theta } - \theta )/\sqrt {\hat{V}(\hat{\theta })}$ follows as approximately standard normal distribution. Therefore, the two-sided $100(1 - \alpha )\,\%$ ACIs for $\theta_{0} ,\theta_{1} ,\theta_{2} ,\lambda$ are given by

$$\left[ {\hat{\theta }_{j} - z_{\alpha /2} \sqrt {\hat{V}_{(j + 1)(j + 1)} } ,\;\;\hat{\theta }_{j} + z_{\alpha /2} \sqrt {\hat{V}_{(j + 1)(j + 1)} } } \right],\quad j = 0,1,2,$$

$$\left[ {\hat{\lambda } - z_{\alpha /2} \sqrt {\hat{V}_{44} } ,\;\hat{\lambda } + z_{\alpha /2} \sqrt {\hat{V}_{44} } } \right],$$

where $z_{\alpha /2}$ is the $\alpha /2$ quantile of a standard normal distribution.

### Bootstrap sample

Step1. Given $n,\;m,\;\tau$ and progressive censoring scheme $(r_{1} , \ldots ,r_{m} )$, compute the MLEs $\hat{\theta }_{0} ,\hat{\theta }_{1} ,\hat{\theta }_{2} ,\hat{\lambda }$ based on the original Type-I progressively hybrid censored sample $(x_{1} , \ldots ,x_{m} )$.

Step2. Based on $n,\;m,\;\tau ,\;(r_{1} , \ldots ,r_{m} )$, $\hat{\theta }_{0} ,\hat{\theta }_{1} ,\hat{\theta }_{2} ,\hat{\lambda }$, generate a Type-I progressively hybrid censored sample $(x_{1}^{*} , \ldots ,x_{m}^{*} )$.

a1. Generate a random sample $w_{1} , \ldots ,w_{m}$ from Uniform distribution $U(0,1)$, where $w_{1} , \ldots ,w_{m}$ are order statistics. Let $v_{l} = w_{l}^{{1/(l + r_{m} + r_{m - 1} + \cdots + r_{m - l + 1} )}}$, $U_{l} = 1 - v_{m} v_{m - 1} \cdots v_{m - l + 1} ,\;\;l = 1,2, \ldots ,m$ are order statistics followed Uniform distribution $U(0,1)$.

a2. We obtain the failures $r$ before time $\tau$ and the terminal time $\tau^{*}$.

If $$U_{m} \le 1 - \exp \{ - ((\hat{\theta }_{0} + \hat{\theta }_{j} )/\hat{\lambda })(e^{{\hat{\lambda }\tau }} - 1)\}$$, $$r = m$$, $$\tau^{*} = (1/\hat{\lambda })\ln [1 - (\hat{\lambda }/(\hat{\theta }_{0} + \hat{\theta }_{j} ))\ln (1 - U_{m} )]$$;

If $U_{m} > 1 - \exp \{ - ((\hat{\theta }_{0} + \hat{\theta }_{j} )/\hat{\lambda })(e^{{\hat{\lambda }\tau }} - 1)\}$, $r = J$, $\tau^{*} = \tau$, where *J* is obtained from the inequality

$U_{J} < 1 - \exp \{ - ((\hat{\theta }_{0} + \hat{\theta }_{j} )/\hat{\lambda })(e^{{\hat{\lambda }\tau }} - 1)\} \le U_{J + 1}$, for $1 \le l \le r$, we set $x_{l}^{*} = (1/\hat{\lambda })\ln [1 - (\hat{\lambda }/(\hat{\theta }_{0} + \hat{\theta }_{j} ))\ln (1 - U_{l} )]$.

Step3. Based on $n,\;m,\;r,\;\tau^{*} ,\;(r_{1} , \ldots ,r_{r} )$ and $(x_{1}^{*} , \ldots ,x_{r}^{*} )$, we obtain the MLEs $\hat{\theta }_{0}^{*} ,\hat{\theta }_{1}^{*} ,\hat{\theta }_{2}^{*} ,\hat{\lambda }^{*}$.

Step4. Repeat steps 2–3 *N* times, we obtain *N* estimates $\left\{ {\hat{\theta }_{j}^{*(i)} ,\hat{\lambda }^{*(i)} } \right\}\;\;(i = 1,2, \ldots ,N;j = 0,1,2)$. Arrange them in ascending order to obtain the bootstrap sample $\left\{ {\hat{\theta }_{j}^{*(1)} ,\hat{\theta }_{j}^{*(2)} , \ldots ,\hat{\theta }_{j}^{*(N)} ;\;\;\hat{\lambda }^{*(1)} ,\hat{\lambda }^{*(2)} , \ldots ,\hat{\lambda }^{*(N)} } \right\},\;\;j = 0,1,2$.

The two-sided $100(1 - \alpha )\%$ percentile bootstrap confidence intervals (Boot-P CIs) for parameters $\theta_{0} ,\theta_{1} ,\theta_{2} ,\lambda$

$$\left( {\hat{\theta }_{0L}^{*} ,\hat{\theta }_{0U}^{*} } \right) = \left( {\hat{\theta }_{0}^{*(N\alpha /2)} ,\hat{\theta }_{0}^{*(N(1 - \alpha /2))} } \right),\;\left( {\hat{\theta }_{1L}^{*} ,\hat{\theta }_{1U}^{*} } \right) = \left( {\hat{\theta }_{1}^{*(N\alpha /2)} ,\hat{\theta }_{1}^{*(N(1 - \alpha /2))} } \right)$$

$$\left( {\hat{\theta }_{2L}^{*} ,\hat{\theta }_{2U}^{*} } \right) = \left( {\hat{\theta }_{2}^{*(N\alpha /2)} ,\hat{\theta }_{2}^{*(N(1 - \alpha /2))} } \right),\;\left( {\hat{\lambda }_{L}^{*} ,\hat{\lambda }_{U}^{*} } \right) = \left( {\hat{\lambda }^{*(N\alpha /2)} ,\hat{\lambda }^{*(N(1 - \alpha /2))} } \right).$$

## Simulation and data analysis

### Simulation

In this section, we presented some simulation results to evaluate the performance of all the methods proposed in the previous sections for different sample size *n*, different effective sample size *m* and different dependence structure $\theta_{0}$.

Consider two competing failure modes, the initial values for parameters $(\theta_{1} ,\;\theta_{2} ,\;\lambda )$ are $(1.2,\;1,\;0.6)$. Take the dependence structure $\theta_{0} = 0,\;0.3,\;0.8,1.2,1.6$, where $\theta_{0} = 0$ indicates that the two competing failure modes are independent Generate the Type-I PHC samples from the Gompertz distribution $GP(\lambda ,\theta_{0} + \theta_{j} )$ for competing failure mode $j(j = 1,2)$ according to the algorithm proposed by Balakrishnan and Sandhu (1995). Take the terminal time $\tau = 1$, and *n* = 20, 30, 50, *m* = 4, 6, 8, 10, 15, the pre-fixed scheme $(r_{1} , r_{2} , \ldots , r_{m} )$ are

$$n = 20,\;m = 4,\;r_{1} = r_{2} = \cdots = r_{m} = 4,$$

$$n = 20,\;m = 8,\;r_{1} = r_{2} = 3,r_{3} = r_{4} = \cdots = r_{m} = 1,$$

$$n = 30,\;m = 6,\;r_{1} = r_{2} = \cdots = r_{m} = 4,$$

$$n = 30,\;m = 10,\;r_{1} = r_{2} = \cdots = r_{m} = 2,$$

$$n = 50,\;m = 10,\;r_{1} = r_{2} = \cdots = r_{m} = 4,$$

$$n = 50,\;m = 15,\;r_{1} = 5,r_{2} = 4,r_{3} = r_{4} = \cdots = r_{m} = 2.$$

**Fig. 2:**
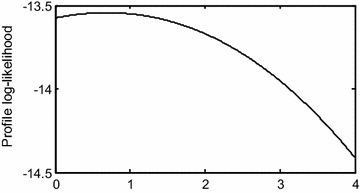
Profile log-likelihood function of *λ*

To compute the MLEs of $\lambda$, we have used the iterative procedure described in “Maximum likelihood estimations (MLEs)” section and stopped the iterative procedure when the difference between two consecutive iterates is less than $10^{ - 4}$. Before going to compute the MLEs, we plot the profile log-likelihood function of *λ* in Fig. 2. Figure 2 shows that the profile log-likelihood function of *λ* is unimodal, the MLE of *λ* is close to 0.6, so we start the iteration with the initial guess that $\lambda^{(0)} = 0.6$.

Repeat 10,000 times for each given *n*, *m*, $\theta_{0}$ and censoring scheme, the average mean squared errors (MSEs) and the average absolute relative bias (RABias) and the coverage percentage of the ACIs and Boot-P CIs are shown in Tables 1, 2 and 3.

**Table 1:** *n* = 20, $$1 - \alpha = 0.95$$

*m*	*θ*_0_	*θ*_0_		*θ*_1_		*θ*_2_		*λ*	
		MSEs	ACI	MSEs	ACI	MSEs	ACI	MSEs	ACI
		RABias	Boot-P	RABias	Boot-P	RABias	Boot-P	RABias	Boot-P
4	0	0.221	0.963	0.8171	0.900	0.7271	0.912	0.2516	0.904
			0.908	0.7144	0.869	0.837	0.836	0.7974	0.902
	0.3	0.1299	0.958	0.7194	0.914	0.6848	0.857	0.2402	0.980
		0.7855	0.934	0.6744	0.877	0.7961	0.852	0.7832	0.934
	0.8	0.4077	0.969	0.626	0.929	0.6395	0.940	0.2315	0.965
		0.7278	0.886	0.6189	0.907	0.7621	0.917	0.7672	0.978
	1.2	0.9094	0.974	0.6771	0.927	0.629	0.931	0.2245	0.963
		0.7431	0.896	0.5798	0.912	0.7307	0.923	0.7516	0.986
	1.6	1.7076	0.961	0.7727	0.918	0.639	0.915	0.2184	0.947
		0.7724	0.866	0.5649	0.905	0.7023	0.891	0.7409	0.991
8	0	0.1081	0.925	0.4369	0.899	0.5514	0.903	0.217	0.941
			0.898	0.5018	0.879	0.704	0.853	0.7241	0.897
	0.3	0.1057	0.957	0.3504	0.915	0.4752	0.947	0.215	0.949
		0.7145	0.925	0.4321	0.891	0.6533	0.869	0.7125	0.924
	0.8	0.2489	0.952	0.3168	0.957	0.4051	0.974	0.2038	0.987
		0.5349	0.967	0.3832	0.934	0.5686	0.967	0.6984	0.954
	1.2	0.5723	0.917	0.3634	0.905	0.365	0.943	0.1952	0.942
		0.563	0.968	0.3784	0.951	0.5278	0.977	0.6822	0.952
	1.6	1.1113	0.906	0.5161	0.899	0.3765	0.902	0.1962	0.937
		0.6053	0.879	0.4238	0.893	0.5122	0.904	0.6882	0.936

**Table 2:** *n* = 30, $$1 - \alpha = 0.95$$

*m*	*θ*_0_	*θ*_0_		*θ*_1_		*θ*_2_		*λ*	
		MSEs	ACI	MSEs	ACI	MSEs	ACI	MSEs	ACI
		RABias	Boot-P	RABias	Boot-P	RABias	Boot-P	RABias	Boot-P
6	0	0.1572	0.907	0.6745	0.907	0.6719	0.898	0.2554	0.933
			0.903	0.6554	0.911	0.7931	0.895	0.7989	0.892
	0.3	0.0848	0.958	0.5776	0.898	0.6121	0.914	0.2484	0.929
		0.6838	0.921	0.5969	0.899	0.753	0.875	0.7953	0.928
	0.8	0.3152	0.971	0.4578	0.957	0.5416	0.971	0.239	0.968
		0.6385	0.868	0.517	0.897	0.6897	0.901	0.7812	0.943
	1.2	0.7678	0.980	0.4317	0.968	0.5079	0.934	0.234	0.988
		0.6835	0.937	0.4716	0.913	0.6526	0.915	0.7744	0.983
	1.6	1.5004	0.929	0.4752	0.918	0.4819	0.927	0.2291	0.951
		0.728	0.898	0.4655	0.826	0.6298	0.877	0.7648	0.889
10	0	0.1101	0.917	0.4546	0.913	0.5544	0.899	0.2253	0.914
			0.914	0.5234	0.895	0.7262	0.879	0.7441	0.927
	0.3	0.0733	0.929	0.3553	0.924	0.485	0.908	0.2227	0.931
		0.6668	0.920	0.4471	0.894	0.6565	0.897	0.7432	0.930
	0.8	0.2195	0.972	0.2674	0.961	0.3879	0.962	0.2173	0.947
		0.5113	0.939	0.3632	0.869	0.5776	0.929	0.7338	0.946
	1.2	0.5649	0.968	0.2733	0.977	0.3314	0.984	0.2108	0.967
		0.5739	0.965	0.3404	0.915	0.513	0.978	0.7224	0.938
	1.6	1.135	0.943	0.3163	0.929	0.3108	0.953	0.2088	0.905
		0.6222	0.865	0.3525	0.886	0.4856	0.912	0.72	0.894

**Table 3:** *n* = 50, $$1 - \alpha = 0.95$$

*m*	*θ*_0_	*θ*_0_		*θ*_1_		*θ*_2_		*λ*	
		MSEs	ACI	MSEs	ACI	MSEs	ACI	MSEs	ACI
		RABias	Boot-P	RABias	Boot-P	RABias	Boot-P	RABias	Boot-P
10	0	0.1443	0.916	0.5431	0.931	0.6052	0.909	0.25	0.933
			0.897	0.588	0.865	0.7577	0.842	0.794	0.934
	0.3	0.0577	0.914	0.4382	0.948	0.535	0.914	0.2467	0.941
		0.5855	0.897	0.5133	0.878	0.7088	0.845	0.7933	0.928
	0.8	0.2409	0.929	0.311	0.961	0.4439	0.968	0.2375	0.948
		0.5498	0.935	0.4103	0.920	0.627	0.896	0.7867	0.956
	1.2	0.6486	0.968	0.2746	0.967	0.3905	0.955	0.2349	0.967
		0.6286	0.963	0.3673	0.936	0.5754	0.911	0.7782	0.967
	1.6	1.2801	0.941	0.2878	0.949	0.3568	0.947	0.2311	0.958
		0.672	0.855	0.3549	0.864	0.5369	0.866	0.7719	0.936
15	0	0.1052	0.928	0.396	0.937	0.5194	0.934	0.2193	0.928
			0.892	0.4954	0.899	0.6969	0.802	0.7281	0.927
	0.3	0.051	0.933	0.2819	0.946	0.4332	0.929	0.2161	0.941
		0.5615	0.921	0.4028	0.878	0.6385	0.863	0.7245	0.936
	0.8	0.1705	0.967	0.1874	0.968	0.3368	0.964	0.2095	0.973
		0.4524	0.959	0.298	0.936	0.5408	0.938	0.722	0.957
	1.2	0.4865	0.972	0.1725	0.971	0.282	0.978	0.2068	0.968
		0.5337	0.951	0.2746	0.941	0.4781	0.942	0.7157	0.949
	1.6	1.0174	0.928	0.2367	0.944	0.2386	0.929	0.2048	0.927
		0.5948	0.836	0.302	0.855	0.424	0.914	0.7125	0.934

From Tables 1, 2 and 3, the observations can be made. For fixed sampling scheme, sample size *n* and dependence structure $\theta_{0}$, the MSEs and RABias decrease as the effective sample size *m* increase.

For fixed sampling scheme, sample size *n* and effective sample size *m*, as the dependence structure of competing failure modes become stronger, the MSEs and RABias get smaller, while the MSEs and RABias with $\theta_{0} = 0$ are bigger, which shows that the performance of the MLEs depends on the strength of dependence. This also shows that the dependence structure is very important in the competing risks model.

For fixed sampling scheme, *n*, *m* and dependence structure $\theta_{0}$, the ACIs are stable than the Boot-P CIs, they can maintain their coverage percentages at the pre-fixed normal level.

### Data analysis

**Fig. 3:**
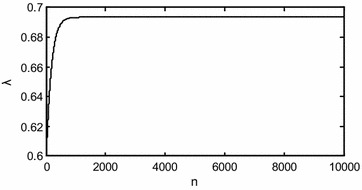
Trace plot of MLE for *λ* using the iterative procedure

Using the procedures above, we generate the Type-I PHC samples when $(n,m,\tau ) = (30,10,1)$ with initial value for parameters $(\theta_{1} ,\;\theta_{2} ,\;\lambda )$ as $(1.2,\;1,\;0.6)$, and the dependence structure $\theta_{0} = \;0.8$, the censoring scheme as $r_{1} = r_{2} = \cdots = r_{m} = 2$. The simulated data is listed in Table 4. The MLEs and 95 % ACIs and Boot-P CIs are shown in Table 5. The trace plot of the MLE for parameter $\lambda$ using the iterative procedure is shown in Fig. 3, which shows that the estimate of $\lambda$ converges to a value after about 1000 iterations.

**Table 4:** The simulated data

*i*	1	2	3	4	5	6	7	8	9	10
$t_{i}$	0.0035	0.0181	0.0435	0.0813	0.0860	0.1286	0.1483	0.1484	0.1929	0.4449
$\alpha_{i}$	2	2	0	0	2	1	0	1	1	2

**Table 5:** MLEs and 95 % CIs of the parameters

Para.	True value	MLE	ACI	Boot-P CI
*θ*_0_	0.8	0.8934	(0.3777, 2.1645)	(0.2811, 0.9764)
*θ*_1_	1.2	0.6627	(0.1987, 1.5241)	(0.1728, 1.3569)
*θ*_2_	1	0.8136	(0.1167, 1.7438)	(0.2718, 1.1114)
*λ*	0.6	0.6935	(0.1911, 2.9921)	(0.4962, 0.7265)

## Conclusion

This paper proposed the dependent competing risks model from Gompertz distribution under Type-I PHCS. We obtained the MLEs and ACIs and Boot-P CIs for the parameters. Simulations showed that the ACIs are more stable than the Boot-P CIs and that the dependence structure is important in the competing risks model. For a given sample size, the performance of the MLEs declined with increasing dependence, which suggests that greater dependence will require a larger sample size to achieve a particular level of precision in estimation.
